# Sustainable materials: a linking bridge between material perception, affordance, and aesthetics

**DOI:** 10.3389/fpsyg.2023.1307467

**Published:** 2024-01-08

**Authors:** Francesca Strappini, Sabrina Fagioli, Stefano Mastandrea, Claudia Scorolli

**Affiliations:** ^1^Department of Philosophy and Communication, University of Bologna, Bologna, Italy; ^2^Department of Education, “Roma Tre” University, Rome, Italy

**Keywords:** sustainability, material property, beauty, affordances, aesthetic appreciation, aesthetics

## Abstract

The perception of material properties, which refers to the way in which individuals perceive and interpret materials through their sensory experiences, plays a crucial role in our interaction with the environment. Affordance, on the other hand, refers to the potential actions and uses that materials offer to users. In turn, the perception of the affordances is modulated by the aesthetic appreciation that individuals experience when interacting with the environment. Although material perception, affordances, and aesthetic appreciation are recognized as essential to fostering sustainability in society, only a few studies have investigated this subject matter systematically and their reciprocal influences. This scarcity is partially due to the challenges offered by the complexity of combining interdisciplinary topics that explore interactions between various disciplines, such as psychophysics, neurophysiology, affective science, aesthetics, and social and environmental sciences. Outlining the main findings across disciplines, this review highlights the pivotal role of material perception in shaping sustainable behaviors. It establishes connections between material perception, affordance, aesthetics, and sustainability, emphasizing the need for interdisciplinary research and integrated approaches in environmental psychology. This integration is essential as it can provide insight into how to foster sustainable and durable changes.

## Introduction

In today’s rapidly evolving world, the interconnectedness of human behavior, material perception, and sustainability have become increasingly important. From a psychological point of view, understanding how we perceive and interact with the materials around us, as well as the *tertiary qualities* (Koffka, 1940; Sinico, 2015) or “affordances” they offer, may play a crucial role in shaping sustainable practices and design strategies. This intricate relationship between human perception and material affordances forms the core of our exploration into the concept of affordance.

Coined by the environmental psychologist Gibson (1977), affordance refers to the perceived possibilities for *action* that an object or environment offers. It suggests that our perception of materials goes beyond physical attributes, extending to the potential actions they enable or constrain. Gibson stated, “I assume that affordances are not simply phenomenal qualities of subjective experience … instead, they are ecological, in the sense that they are properties of the environment relative to an animal” (Gibson, 1966, p. 285). This concept was highly influenced by the Gestalt theory and prefigured by the work of Lewin and Koffka, who stressed the dynamic and functional relationship between environmental objects and what a perceiving and acting organism can do (Buxton, 1985) and highlighted the inter-subjective character of tertiary qualities (Koffka, 1940). In this context, tertiary qualities, for example, the “expressive” or “physiognomic” properties, are functional qualities that express a particular (moral, psychological, intersensory) character (e.g., velvet is kind) and, although they need the activity of an organism to perceive them (through the nervous system), they are independent of the subject (Koffka, 1940, p. 191). As the concept of *affordance* has been used in many different contexts and research fields, with slight variations in meaning, here we define affordance for a given function as the perception of the “how-to-use it” when seeing the object or when the surface correlates with the structure (functionalism). Moreover, we adhere to the defining criteria proposed by Evans et al. (2017): (i) it is neither the object nor a feature of the object; (ii) it is not an outcome; (iii) it has variability.

Material perception comes into play as we interpret and understand the qualities and properties of different materials through our senses, particularly vision and touch. Materials’ texture, weight, and temperature provide vital information about their composition and potential uses. This perceptual understanding influences how we interact with materials and make decisions regarding their sustainable usage.

This review addresses environmental sustainability challenges, traditionally tackled with technological innovations, emphasizing a shift in paradigms and theoretical frameworks that consider people’s attitudes, values, beliefs, and emotional needs. As awareness grows about the environmental impact of our actions, there is a growing need to consider the affordances of sustainable materials, aesthetic appreciation, and practices. Designers and innovators are exploring ways to create materials and products that meet our functional needs and align with ecological considerations. Hence, this review aims to examine the relationship between surface material perception (as the predictor) and sustainable actions (as the outcome), exploring the potential mediating roles of object affordance and aesthetic appreciation, encompassing research on beauty, aesthetic pleasure, and preference. Understanding the functional and aesthetic aspects of sustainable materials fosters thoughtful and environmentally conscious consumption choices by increasing the perceived object’s value and lifespan.

In the following sections, we will begin by discussing the psychophysics and neural correlates of material perception. Next, we will delve deeper into the intricate relationship between material perception, affordance, and sustainability, exploring how these concepts interact and influence one another. Additionally, we will explore the connection between sustainable materials and aesthetic appreciation. Our goal is to bridge disciplinary gaps, bringing together fields that typically operate independently. This exploration enhances our understanding of these interconnected phenomena and contributes to fostering environmentally conscious choices and sustainable behaviors.

## Visual psychophysics of material perception

Material perception is how we perceive what things are made of, the material composition of objects. Although this function may encompass all our senses, we will focus on how material categories and properties are extracted from the visual environment.

When tested using high-level material categories and real-world images, it has been shown that visual recognition of materials is a rapid process despite the visual ambiguity, where similarities in visual appearance are found across different types of materials and variations in visual appearance are found within a single type of material (Sharan et al., 2009, 2014). In a study by Sharan et al. (2009), the participant’s task was to identify the material types in photographs taken in the real world. The authors used photographs from the Flickr.com material image database to test material detection in a rapid serial visual presentation (RSVP) paradigm (Sharan et al., 2009). The results showed that observers could still complete the recognition task even with stimulus display times as brief as 40 ms. The recognition was not based on other cues such as object recognition, shape, texture, and color discrimination (Sharan et al., 2009; Scorolli and Borghi, 2015). These results are consistent with a more recent study that has shown that the categorization of materials is accurate but slower than object recognition (Wiebel et al., 2013). Using a backward-masking paradigm, the authors looked at how material categorization in natural photographs changes over time in relation to superordinate and basic-level object categorization. The findings demonstrated that the speed of material categorization is slower than that associated with superordinate object categorization but generally equivalent to the speed of basic-level object categorization. Subjects’ performance was modulated by color, which significantly increased performance for material categorization, suggesting that low-level features are crucial in mediating performance. Although modulated by low-level features, material recognition seems to occur at a higher stage of the visual hierarchy compared to the processing of low-level features such as color, motion, and orientation. Indeed, Wolfe and Myers (2010) have shown, with a visual search paradigm, that material type is associated with inefficient search results, suggesting that this attribute does not guide our visual search in the visual environment, and it is probably challenged by the visual phenomenon of “crowding” presented in the peripheral vision (Pelli et al., 2004; Strappini et al., 2017; Wolfe and Horowitz, 2017).

A recent theoretical framework, namely, the “statistical appearance models,” has been proposed by Fleming (2014, 2017) to explain material recognition. This model presupposes the existence of a high-dimensional feature space, and it uses generative models that are specific for encoding and recognizing materials. This paradigm proposes that rather than learning the fundamental physical laws of the outside world, we learn to encode the systematic changes related to low-level attributes such as size and contrast, both within and between materials. For this reason, this framework may explain why the judgment of a certain property surface material (e.g., gloss) is strongly influenced by the judgment of another surface attribute, as perceivers seem to compare the relative salience of segmented parts when they are asked to judge the material properties (Fleming, 2014).

## Neural correlates of material perception

There is a general agreement in considering material perception as a mid-stage, cross-modal process with a hierarchical structure in terms of visual perception. However, its neural basis and how material properties are encoded on a neuronal or network level it is not clearly understood. Functional magnetic resonance (fMRI) studies in humans have found that visual material processing is associated with the blood oxygenation level-dependent (BOLD) activity in the medial regions of the ventral extrastriate cortex (e.g., Newman et al., 2005; Cant and Goodale, 2007, 2011; Jacobs et al., 2014) and the high-order visual areas, such as the parahippocampal gyrus, fusiform gyrus, and collateral sulcus (Hiramatsu et al., 2011; Goda et al., 2014; Komatsu and Goda, 2018). Only a few studies have investigated the neural processing associated with processing specific material properties. For instance, Sun et al. (2016) have found that surface properties significantly modulate the activity in the early visual and somatosensory cortex. Gloss, which is one of the most studied material properties, seems to be associated with neural activity in the posterior fusiform sulcus and in area V3B/KO (Sun et al., 2015) in humans and the inferior temporal cortex in monkeys (Nishio et al., 2012; Baba et al., 2021).

Hiramatsu et al. (2011) performed an fMRI experiment to examine how the human brain categorizes material categories utilizing multivoxel pattern analysis. They showed that low-level image statistics, including contrast, spatial frequency, and color information, greatly influence how materials are represented in the early visual areas. This result is consistent with a recent study that showed that roughness and texturedness could be classified based on image statistics as early as the striate cortex, and therefore, that category information is already present in V1 (Baumgartner and Gegenfurtner, 2016). These results are consistent with an event-related potentials (ERPs) study showing that material categories, such as wood and stone, can be discriminated systematically around 100 ms after stimulus onset, probably due to differences in the low-level image attributes between the surface material properties (Wiebel et al., 2014).

Overall, these neuroimaging studies seem to suggest that the neural processing in material perception may range from identifying basic image features in the primary and secondary visual cortex (Baumgartner and Gegenfurtner, 2016) to classifying surface materials in higher-order category areas, such as the parahippocampal gyrus, fusiform gyrus, and collateral sulcus (Hiramatsu et al., 2011; Goda et al., 2014; Jacobs et al., 2014; Komatsu and Goda, 2018).

At the clinical level, this pattern of results is consistent with neuropsychological studies showing a dissociation between shape, size, and orientation processing, which seem more related to the occipitotemporal portion of the lateral occipital complex (LOC) and the material properties processing, which seems more associated with the lateral subdivision of LOC (James et al., 2003).

## Active perception and material affordance

The information gained through material perception can be used to appropriately control the movements of one’s fingers when grasping objects or one’s feet when walking on a road or deciding whether to purchase a product. These functions are performed thanks to learning the relationship between object attributes and effective object interaction.

Among object categories like faces, body parts, animals, houses, and scenes, tools automatically engage “unconscious” sensorimotor modules and corresponding cortical regions in the posterior parietal cortex (the “dorsal stream”) even during passive viewing (e.g., Creem-Regehr and Lee, 2005; Kourtis et al., 2018; Whitwell et al., 2020). This process is similar to the motor “affordance” phenomenon, which occurs when the perception of a graspable object prompts motor actions that are consistent with the object’s orientation or size (Tucker and Ellis, 1998, 2001; Ellis and Tucker, 2000; Phillips and Ward, 2002). Clinically, patients with manual groping or utilization behavior that forces the patient’s hand to follow, grip, or utilize tools can exhibit the automatic nature of uninhibited motor affordances (Lhermitte, 1983).

The enhancement of an action provided by an object may happen automatically (Tucker and Ellis, 1998). However, it also depends on several factors, including the attention given to the object as a whole (Riggio et al., 2008) or to an action-relevant feature of the object (Pellicano et al., 2010), the shaping of the prospective individual’s hands (Ansuini et al., 2008), the actual possibility of reaching the object (Cardellicchio et al., 2011), the parallel linguistic processing (Ambrosini et al., 2012), the possible social request from a conspecific (Scorolli et al., 2014), as well as the involvement of the affective dimension (Caravà and Scorolli, 2020). The affordances of an object can also vary based on the non-permanent attributes of an object and its time-invariant features (Borghi and Riggio, 2009, 2015).

Few studies have examined the affordances related to material properties and whether changes in material surface appearance affect motor movements of prehension (reaching and grasping). Grasping involves preparing the grip by opening and shutting the hand according to the desired object’s characteristics, whereas reaching involves directing the hand to the desired position (Jeannerod, 1981, 1984).

Paulun et al. (2016) investigated how material properties and object orientation influence precision grip kinematics. They gave participants cylinders to hold, raise, and carry to a specific location, which were composed of different materials (styrofoam, wood, brass, and a vaseline-coated) and displayed at six distinct orientations (0°, 30°, 60°, 90°, 120°, and 150°) in relation to the participants. Differences in time and spatial modulation at all stages of the movement were found by analyzing their grasping kinematics, which depended on both material and orientation. Specifically, the material had an impact on the selection of local grasp locations as well as the length of the movement from the initial visual input to the object’s release (Paulun et al., 2016).

A recent study employed a seated reach-to-grasp paradigm, where participants performed a lifting movement transporting familiar objects, paper cups, from one location to another, varying the surface glossiness and object weight. The authors found that the temporal and spatial components of the reach-to-grasp movements were modulated not only by the weight, as previously shown, but also by variations in the surface material properties (matte vs. varnished surface) (Ingvarsdóttir and Balkenius, 2020). In a follow-up study, the authors investigated how material properties influence the early grip force control exerted by each of the five fingers while lifting paper cups (Ingvarsdóttir and Balkenius, 2020). As in the previous study, object weight and surface glossiness were modulated across conditions. The outcomes confirmed the importance of visual material qualities in prehension control. Moreover, it was shown that early grip force scaling was affected not only by the weight of the cups but also by their surface glossiness (Ingvarsdóttir and Balkenius, 2020).

Finally, one study examined the development of material property perception for grasping and reaching in early childhood for objects with different rigidity using a 3D motion capture system (Preißler et al., 2021). The task consisted in lifting objects with one of two handles that varied in rigidity (soft and hard) after visual and visual-haptic exploration. The findings showed that after visual exploration, infants had no specific material preference; thus, the material did not ease grasping. However, after visual-haptic exploration, the infants preferred the soft handles, although they were more challenging to use when lifting the object. Conversely, adults showed the opposite pattern as they preferred using rigid handles to grasp the object and were efficient with both conditions. Interestingly, 3 years-old children seemed to be in an in-between stage of development and showed no preference for the soft or rigid type of handle. These results suggest that reaching and grasping objects is influenced by the material property, such as rigidity, and that it is a learned skill that requires a long development process (Purpura et al., 2018), as the efficient use of visual and visual-haptic information presumably appears later than the age of 3 years.

## Aesthetic sustainability of materials perception

How individuals perceive materials significantly influences their decisions and actions towards sustainable practices and design choices. Understanding the determinants of material perception is important for promoting environmentally conscious behaviors and informing sustainable design practices.

Recent research has highlighted the relationship between material perception and sustainable decision-making, emphasizing the importance of sensory experiences in driving environmentally conscious choices. Studies have shown that individuals’ perception of materials can influence their willingness to engage in sustainable practices. For example, research by Bjelkemyr et al. (2015) found that participants who perceived materials as more environmentally friendly in terms of life cycle assessment were more likely to think about pro-environmental behaviors, such as recycling and energy conservation. Specifically, the authors found that metals are considered the most important materials to recycle, while plastics are within the waste fractions. This suggests that a positive perception of sustainable materials can contribute to a greater motivation for sustainable action. Furthermore, materials’ tactile and visual qualities can significantly impact their perceived sustainability. Research by Thundathil et al. (2023) showed that when individuals interacted with materials that were visually and haptically associated with sustainability (biobased composites), they exhibited a greater likelihood of attributing eco-friendly characteristics to these materials in terms of beauty, naturality, and value. This association between sensory experiences and sustainability perception highlights the potential for utilizing materials with sustainable attributes to enhance positive perceptions and promote sustainable choices.

On the other hand, other studies have highlighted the importance of the type of material for the perception of sustainability in packaging and how this relationship is associated with eco-friendly choices. For instance, de Oliveira et al. (2023) showed that the perception of sustainability and environmental value was higher when the packaging used materials such as paperboard and glass. Conversely, materials like metals and polymers undermine the perception of this value.

Although product designers are eager to promote sustainable materials, how users feel about them and how material properties interact with sustainability perception still needs to be determined. For instance, bio-plastics are only now available in relatively few niche markets. However, it is still uncertain how consumers would react (Brockhaus et al., 2016). It appears appropriate to look into how people interact with these materials, given the progressive development of sustainable materials by various product developers. When users interact with sustainable materials, their distinctive surface material properties and the “ingredients” that the materials are built of will operate as active cognitive stimuli and, as a result, trigger a range of emotions. In this regard, research on the mediating role of affective responses and emotions on the relationship between material perception and sustainable actions has the potential to inform the development of innovative sustainable materials and products.

A study by Bahrudin and Aurisicchio (2018) found that participants’ evaluation of sustainable materials induced various positive and negative emotions. In particular, the most frequent positive and negative emotions were surprise and disgust, respectively. When the materials were appraised in terms of sustainability and lifecycle parameters, they were perceived as more connected to positive emotions than when they were appraised based on their technical themes or sensorial properties. Thus, the authors conclude that systemic appraisals, for instance, based on the lifecycle assessment of the material, have the benefit of impacting product use. These findings highlight the importance of the narrative, or “biography,” of a sustainable material that can potentially amplify positive emotions. Self-positive and moral emotions also play a role in sustainable perception, in particular positive emotions that promote happiness, health, and quality of life, feeling morally righteous in relation to the environment, and feeling powerful by an increase in the social status (Hain, 2017). Positive emotions are also associated with developing an emotional attachment to the product, which ultimately induces more frequent use and helps extend its lifecycle (Wu et al., 2021). Given that positive emotions are associated with sustainability, several theories have been proposed to explain the relationship between product design and emotions. For instance, the emotionally durable design (EDD) is a method proposed by Chapman (2012) to enhance emotional processing and thus extend a product’s lifecycle.

Several studies suggested the importance of creating customer loyalty inducing emotional attachment with sustainable products and eco-friendly practices. Indeed, it has been found that individuals who feel emotionally connected to sustainable materials are more likely to engage in sustainable behaviors and express a greater willingness to pay for sustainable products (e.g., Laroche et al., 2001; Han et al., 2010). This emotional attachment can be fostered through design strategies that evoke positive sensory experiences, such as using materials with pleasing textures and visual aesthetics that evoke nature or sustainability values. In the realm of design and innovation, the perception of attractive materials can drive sustainable practices. Indeed, aesthetic appreciation, related to materials’ sensory and emotional appeal, seems to influence affordance and sustainable choices. Aesthetically pleasing designs evoke positive emotional responses, increasing product satisfaction and longer product lifespans. Research in this field has focused on finding the best strategy to improve emotional durability and consumers’ aesthetic appreciation of the product (Ji and Lin, 2022). Although the aesthetic appreciation of materials is considered essential to induce sustainable actions, only a few studies have investigated this relationship. A study exploring the perception of the beauty of materials-derived waste based on visual and tactile stimulation found that modifying visual and tactile properties may shift how individuals perceive material aesthetics. Specifically, the authors suggest that for introducing an unfamiliar material, changing the perceptual properties in an incongruent, contrasting way might be a possible strategy to elicit a positive emotion of surprise and, ultimately, appreciation (Sauerwein et al., 2017).

Finally, some studies within the environmental psychology of building design have highlighted the meaningful impact of naturalness, i.e., how a product has a natural-looking aspect, on aesthetic evaluation. In seeking to quantify the low-level features and visual statistics underlying natural-looking environments, researchers have found that the high frequency of contrast changes and high density of curved edges predict aesthetic appreciation (Berman et al., 2014; Kardan et al., 2015). A recent study has found that scaling and contrast patterns (Alexander et al., 2004) are associated with the perception of naturalness and predict the aesthetic preference in interior and exterior architectural images (Coburn et al., 2019). These results suggest that aesthetic preference for naturalistic architectures, regardless of the types, is mediated by a common mechanism. Further studies have also shown that these perceptual mechanisms are shared among non-professionals and professionals, such as architects, as both have the same accuracy in evaluating how a material has a natural look, thus suggesting the importance of maintaining the naturalness of the surface materials in building sustainable products (Zhang et al., 2023). Thus, understanding how we see materials is not just about vision; it guides us toward sustainable choices and eco-friendly designs.

## Concluding remarks

Positive perceptions of sustainable materials, driven by sensory experiences, emotional connections, and visual aesthetics, can influence sustainable decision-making and foster environmentally conscious behaviors leading toward “aesthetic sustainability” (Harper, 2018) or “echo-aesthetics.” Understanding the psychophysics and neural basis of material perception, as a low-mid level phenomenon in the visual processing hierarchy, and its emotional and aesthetic experience can reshape our understanding of the aesthetic and artistic concepts associated with material design and inform research within the field of environmental psychology. Indeed, integrating sustainable materials into design and innovation practices can leverage these perceptions to encourage the adoption of sustainable products.

The present work highlights how the current literature supports the link between material perception, affordance, aesthetics, and sustainability. We believe that this frontier research deserves a focused and joint effort by researchers from different disciplines, from cognitive sciences to design, toward a rethinking of “flexible objects” where the dimension of sustainability is addressed along with the motor and aesthetic components.

## Author contributions

FS: Writing – original draft, Writing – review & editing. SF: Conceptualization, Writing – review & editing. SM: Conceptualization, Writing – review & editing. CS: Conceptualization, Funding acquisition, Supervision, Writing – review & editing.
